# Cutis Aplasia as a clinical hallmark for the syndrome associated with 19q13.11 deletion: the possible role for *UBA2* gene

**DOI:** 10.1186/s13039-015-0123-x

**Published:** 2015-03-26

**Authors:** Joana B Melo, Alexandra Estevinho, Jorge Saraiva, Lina Ramos, Isabel M Carreira

**Affiliations:** Cytogenetics and Genomics Laboratory, Faculty of Medicine, University of Coimbra, Coimbra, Portugal; CIMAGO - Center of Investigation on Environment Genetics and Oncobiology, Faculty of Medicine, University of Coimbra, Coimbra, Portugal; CNC - IBILI - Center of Neurosciences - Institute for Biomedical Imaging and Life Sciences, Coimbra, Portugal; Medical Genetics Unit, Hospital Pediátrico, Centro Hospitalar e Universitário de Coimbra, Coimbra, Portugal; University Clinic of Pediatrics, Faculty of Medicine, University of Coimbra, Coimbra, Portugal

**Keywords:** 19q13.11 deletion, *UBA2* gene, Cutis aplasia

## Abstract

**Background:**

Wide genome screening through array comparative genomic hybridization made possible the recognition of the novel 19q13.11 deletion syndrome. There are very few cases reported with this deletion, but clinically this condition seems to be recognizable by pre and postnatal growth retardation, microcephaly, developmental delay/intellectual disabilities, speech disturbance, hypospadias (in males) and signs of ectodermal dysplasia and cutis aplasia over the posterior occiput.

**Results:**

Using oligoarray CGH, a 4.6 Mb deletion in 19q13.11q13.12 was detected in a 23 year old female patient that presented clinical features previously associated with 19q13.11 deletion.

**Conclusions:**

Our work reinforces the idea that a region encompassing four zinc finger genes is likely to be responsible for the syndrome, and that the difference in minor clinical manifestation depends on the genes present outside the minimal overlapping region proposed for this syndrome. We also review all cases described in the literature and discuss the correlation between haploinsufficiency of *UBA2* gene and cutis aplasia present in the majority of the patients reported, and its importance as a clinical hallmark of 19q13.11 deletion syndrome, when associated with more common features like developmental delay, microcephaly, speech disturbance and hypospadias in males.

## Background

The development of array comparative genomic hybridization technique (array CGH) greatly improved the detection of cryptic unbalanced rearrangements in mental retardation patients and made possible the identification of novel microdeletion and microduplication syndromes [1].

In 1998, Kulharya *et al.,* reported a cytogenetically visible 19q12q13.1 deletion, in a fetus with intrauterine growth retardation and decreased fetal activity. At the age of 3 years the child presented mental retardation, developmental delay, absence of speech, multiple minor anomalies and cutis aplasia [2]. Eleven years later, Malan *et al.*, identified by array CGH a 19q13.11 microdeletion in three patients who share common clinical features with Kulharya *et al.*’s patient, and proposed the 19q13.11 microdeletion syndrome as a novel clinically recognizable syndrome [3]. In 2009, Schuurs-Hoeijmarkers *et al.* narrowed the critical region responsible for the new syndrome to a 750 kb segment within the 19q13.11 deletion [4]. Recently Forzano *et al.* further refined the critical region and Gana *et al.*, proposed a minimal overlaping region (MOR) of 324 kb encompassing four zinc finger genes [5,6].

A total of 8 mental retardation patients and 1 aborted fetus, carrying the 19q13.11 deletion have been reported until now in addition to two more cases included in the Database of Chromosome Imbalance and Phenotype in Humans using Ensembl Resources (DECIPHER) [2-7]. Despite some phenotypic variability, all these patients presented common features and the deletion of 19q13.11 is proposed as a new clinical recognizable syndrome [3].

Patients with this deletion are characterized by intrauterine and postnatal growth retardation, microcephaly, developmental delay/intellectual disabilities, speech disturbance, slender habitus, feeding difficulties, cutis aplasia over the posterior occiput, signs of ectodermal dysplasia, and genital malformation in males (hypospadias). In this study we report a case of a mentally retarded woman carrier of a 19q13.11 deletion and compare our findings with the ones previously reported in the literature [2-7].

## Results

**Figure 1:**
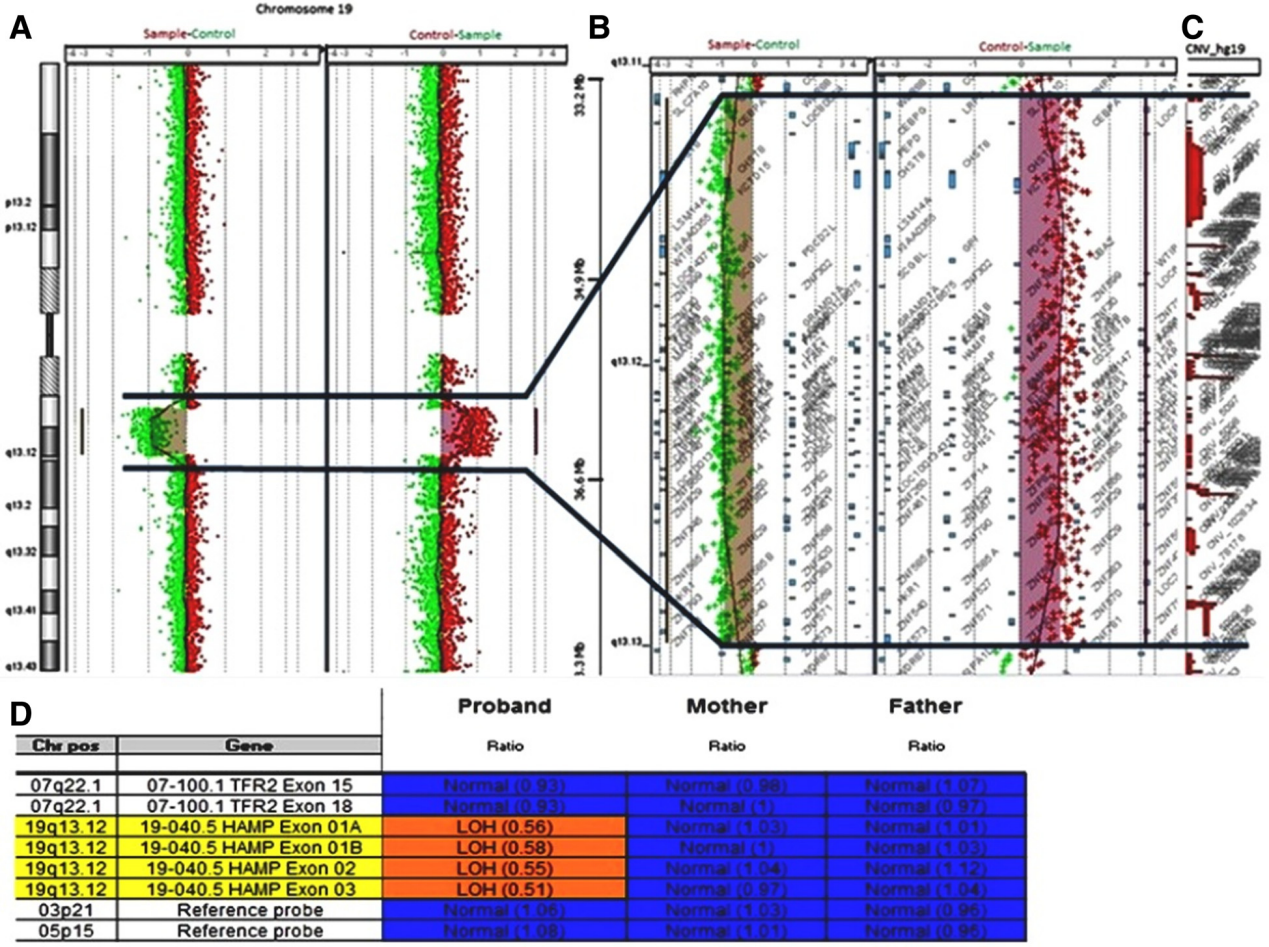
**Molecular cytogenetic analysis. (A)** Array CGH comparative profile for chromosome 19 disclosing the 19q13.12 deletion. **(B)** Zoom in of the deleted region detected by array CGH. **(C)** Zoom in of the CNV profile of the deleted region according to the Database of Genomic Variants (DGV). **(D)** MLPA analysis result of the proband and progenitors (SALSA Probe MLPA – P347-A1) confirming a *de novo* deletion of the probes corresponding to the deletion observed by array CGH.

Patient karyotype was normal 46,XX. Array CGH analysis identified a 4.6 Mb deletion at the long arm of chromosome 19 (Figure 1A-C). The deletion breakpoints were ascertained between 33,203,635 and 38,108,990. Multiplex Ligation-dependent Probe Amplification (MLPA) analysis, using SALSA Probe MLPA – P347-A1 of the proband DNA confirmed the 19q13.11q13.12 *de novo* deletion (Figure 1D). Array CGH final results was arr [hg 19] 19q13.11q13.12 (33,203,635-38,108,990) ×1dn according to the International System for Human Cytogenetic Nomenclature (ISCN) 2013 [8]. The deleted region contains several coding genes, including *CEBPA* [OMIM ID: 116897]*, PEPD* [OMIM ID: 613230]*, LSM14A* [OMIM ID: 610677]*, UBA2* [OMIM ID: 613295]*, WTIP* [OMIM ID: 614790]*, SCGB2B* [OMIM ID:615063]*, ZNF302, ZNF181* [OMIM ID: 606741]*, ZNF599, ZNF30, SCN1* [OMIM ID: 202700], *USF2* [OMIM ID: 600390]*, COX6B1* [OMIM ID: 124089] and *HPHS1.*

## Discussion

Since the Kulharya *et al.* report, of a 3 year-old patient, to the Lin *et al.* report, of an aborted fetus, a total of 8 cases had been reported, in the literature, as examples of the emerging 19q13.11 deletion syndrome [2-7].

**Figure 2:**
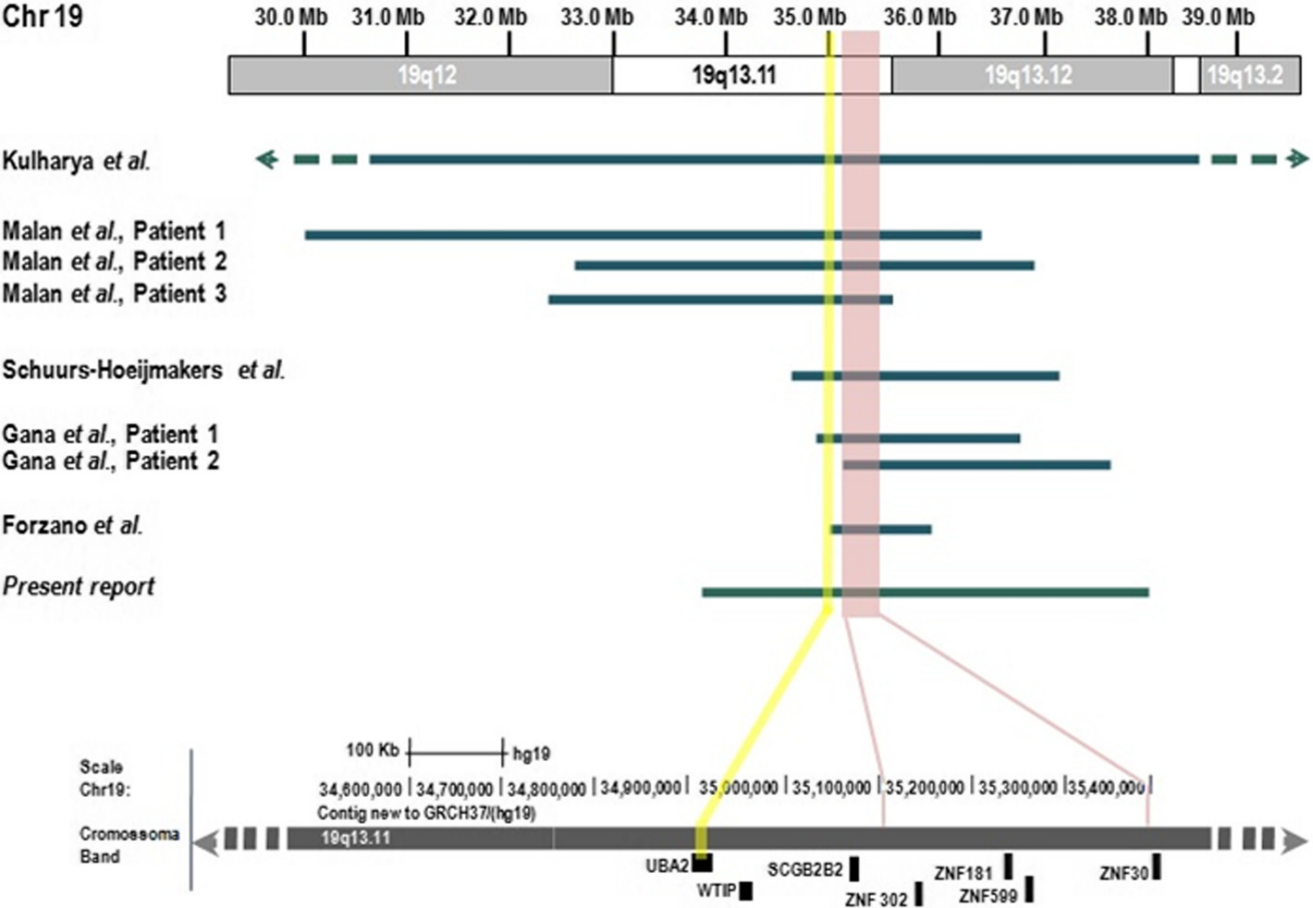
**Schematic representation of the 13.11 deletions at the long arm of chromosome 19.** Representation of eight cases with 19q13.11 deletion, evidencing the MOR region (chr19: 35,111,870-35,436,076) and the genes present. The *UBA2* gene location (GRCh37:34,919,268-34,960,798) is highlighted in yellow.

In this study we identified another patient with a *de novo* 19q13.11q13.12 deletion, harboring the minimal overlapping region (MOR) pointed by Gana *et al.* as the critical region for the 19q13.11 deletion syndrome (Figure 2) [6]. Consistent with typical clinical features in literature [2-7], our case presented intrauterine and postnatal growth retardation, mental retardation, speech disturbance, microcephaly, slender habitus with little subcutaneous fat, facial dysmorphic features, signs of ectodermal dysplasia, cutis aplasia and feeding problems. Comparison of clinical features between our case and the previous reported, evidences that although many anomalies are common they are not all present in all patients (Table 1). The 4.6 Mb deleted region in our patient overlaps the MOR and, contains over 50 coding genes. The specific functions of *ZNF302*, *ZNF181*, *ZNF399* and *ZNF30*, present in the MOR and pinpointed as important genes in this syndrome, have not been completely understood. These zinc finger proteins belong to the *KRAB-ZNF* family, known to be involved in cell differentiation, proliferation, cycle regulation and apoptosis, and in the embryonic development [9,10]. Moreover, the hypothesis that the haploinsuffiency of these genes could be the cause for mental retardation and speech disturbance is supported by the fact that zinc finger genes are involved in X-linked mental retardation in males, and that *ZNF* clusters are suspected to contribute to higher cognitive function in primates [11-14].

**Table 1:** **Clinical features present in patients with 19q13.11 deletion**

	***Kulharya et al.***	***Malan et al.***			***Schuurs- Hoeijmakers et al.***	***Gana et al.***		***Forzano et al.***	***Present report***	***N/T***
		**Patient 1**	**Patient 2**	**Patient 3**		**Patient 1**	**Patient 2**			
**Approximated size of the deletion**	11 Mb	6,2 Mb	4,3 Mb	3 Mb	2,4 Mb	1,7 Mb	2,6 Mb	1,4 Mb	4,9 Mb	
**Gender**	Female	Male	Male	Male	Male	Male	Female	Female	Female	
**Weight at birth**	1,295 Kg	1,560 Kg	1,930 Kg		1,620 Kg	1,590 Kg	1,900 Kg	1,580 Kg	1,950 Kg	
**Developmental characteristics**										
Intrauterine growth retardation	+	+	+	+	+	+	+	+	+	9/9
Postnatal growth retardation	+	+	+	+	+	+	+	+	+	9/9
DD/ID	+	+	+	+	+	+	+	+	+	9/9
Speech disturbance	+	+	+	+	+	+	+	+	+	9/9
Feeding problems	+	+	+	+	+	+		+	+	8/9
Slender habitus	+	+	+	+	+	+	+	+	+	9/9
Microcephaly	+	+	+	+	+	+	+	+	+	9/9
Failure to thrive	+	-	+	-	+	+	+	-	+	6/9
**Facial anomalies**										
Long face	-	+	+	-	-	-	-	+	+	4/9
High forehead	-	+	+	-	-	-	-	-	+	3/9
Micrognathia/Retrognathia	+	+	+	-	+	+	+	+	+	8/9
Low set ears	+	-	+	-	+	-	-	+	+	4/9
V shaped nasal tip	-	+	+	-	-	-	-	-	-	2/9
Thin lips	-	+	+	+	+	+	+	-	+	8/9
**Ectodermal dysplasia**										
Hair/eyebrows/eyelashes anomalies	-	+	+	+	+	+	+	+	+	8/9
Thin/dry skin	-	+	+	-	-	-	-	+	+	4/9
Cutis aplasia in midline scalp	+	+	+	+	+	+	-	+	+	8/9
Dysplastic nails	-	+	+	-	+	-	-	+	+	5/9
Cutaneous syndactyly	-	-	+	+	+	+	-	-	-	4/9
**Extremity abnormalities**										
Clinodactyly	+	+	+	+	+	+	-	+	+	8/9
Overlapping of the toes	+	+	-	-	-	-	-	+	+	4/9
Long fingers	-	+	+	-	+	+	+	-	+	6/9
**Genital abnormalities**										
Hypospadias	Not applicable	+	+	+	+	+	Not applicable	Not applicable	Not applicable	5/5
**Organ abnormalities**										
Congenital heart disease	+	-	+	-	-	+	-	+	+	5/9
Livedo and cutaneous hypersensibility	-	+	-	-	-	-	-	-	+	2/9
Little subcutaneous fat	+	-	+	-	-	-	-	-	+	
Hypotonia	+	-	-	-	-	-	+	-	+	3/9
Dystonia	-	-	-	-	-	+	-	-	+	2/9
Airways infections	+	-	-	+	+	-	-	-	-	3/9

(+) – feature present, (−) – absent feature, DD – development delay, ID – intellectual delay, N/T – number of patients/total of patients.

Neighboring the MOR there are also genes that may be implicated in the syndrome pathogenesis, either because they are also deleted or just because their regulation regions may be disrupted. Schuurs-Hoeijmarkers *et al.*, suggested that the cause of mental retardation in 19q13.11 deletion syndrome could also be associated to haploinsuffiency of genes *LSM14A* and *UBA2* [4]. The *LSM14A* is a Sm-like protein thought to have a role in the control of mRNA translation, and *UBA2* is important in the ubiquitin pathway [4]. Other genes outside the MOR, whose deletions could be associated with major clinical features, are: *CEBPA* gene, as possible cause of lack of subcutaneous fat; *COX6B1* gene, associated with severe infantile encephalomyopathy; and *WT1* and *WTIP* genes that are involved in mammalian urogenital development [15,16].

Cutis aplasia is one of the major features presented by 19q13.11 deletion syndrome patients. However, Gana *et al.* Patient 2, does not present such features (Table 1). Comparing patients 19q13.11 deleted regions, it becomes evident that 34,9 – 35,1 Mb segment is deleted in all patients except in Gana *et al*. Patient 2. This segment harbors three genes: *SCGB2B2, WTIP* and *UBA2*, whose haploinsuffiency could be considered as a cause for cutis aplasia. This hypothesis can be ruled out for *SCGB2B2* since this gene is not expressed in skin cells and is not likely for *WTIP* gene because its haploinsuffiency has been pointed as a cause for hypospadias [5]. Deletions of the *UBA2* gene (codifier of a ubiquitin-like modifier), results into proteins with abnormal post-translational modifications, and in particular could result in prolidase deficiencies. It has been reported that prolidase deficiencies, may impairs proline recycle, causing recurrent cutaneous ulcers that are difficult to heal resulting in cutis aplasia. This could also account for the thin skin, observed in some patients, reinforcing the role for *UBA2* gene in cutis aplasia [2].

Apart from the core phenotype, other clinical features seemed to be associated with genes outside the MOR: hypotonia has been reported and associated with *UQCRFS1* and *VIB* genes; renal anomalies, associated with *HPHS1* and *USF2* genes; and cardiac defects associated with *SCN1B* gene [2-5]. Although these genes are deleted in several cases, patients do not present the same phenotype, leading to suppose that they can act like a predisposing factor that can be trigged under a certain genetic, epigenetic or environmental context.

## Conclusions

In conclusion, 19q13.11 deletion syndrome is a continuous genetic disease where a critical region is responsible for the major clinical features. Other genes outside that region contribute to clinical features observed less frequently. Reviewing all the reported cases and ours we believed that cutis aplasia could be related to haploinsuffiency of *UBA2* gene. The report of further patients with this deletion would reinforce this evidence.

## Methods

### Patient report

**Figure 3:**
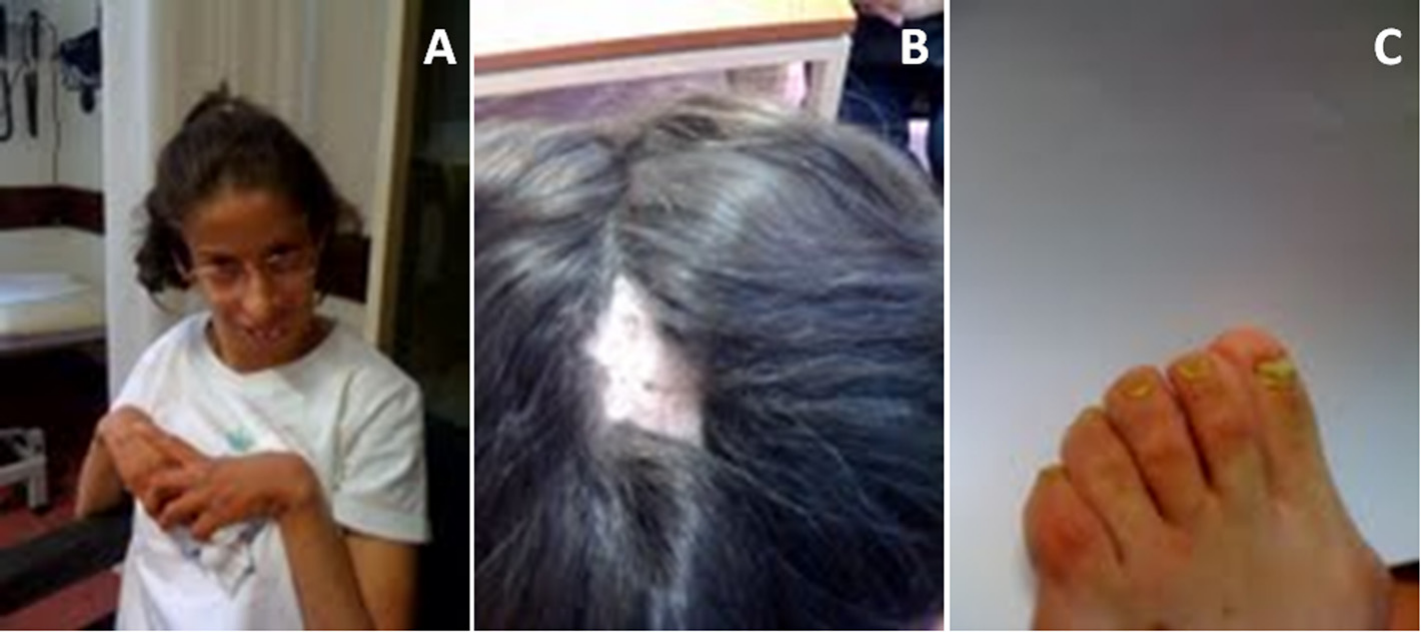
**Dysmorphic features presented by the 23 year old patient. (A)** Photograph of the patient with 23 years old. **(B)** Photograph evidencing cutis aplasia over the posterior occiput. **(C)** Photograph evidencing dysplastic nails.

Our patient was born from non-consanguineous healthy parents, after 39 weeks of gestation and a complicated pregnancy due to intrauterine growth retardation. At birth she weight 1950 g (<5th centile), measured 42 cm (<5th centile) and her head circumference was 30 cm (<5th centile). After birth, she presented feeding difficulties, and it was necessary the presence of a gastrostomy button. Some dysmorphic features were presented, like microcephaly, long face, high forehead, low set ears, deep set eyes, V shaped nasal tip, hipoplasic alae nasi, thin lips, retrognathia and high arched palate. After teething, teeth abnormalities were presented. Signs of ectodermal dysplasia included sparse eyebrows and eyelashes, cutis aplasia in midline scalp, thin and dry skin and dysplastic nails (Figure 3). Extremity abnormalities were noticed, with clinodactyly, long finger and overlapping toes. Other anomalies included congenital heart disease, congenital dislocation of the hip, livedo and cutaneous hypersensibility. Her early years were compromised by feeding difficulties and failure to thrive. From the first (7 month old) to her last examination (23 years old) she always presented slender habitus, she was very thin with very little subcutaneous fat tissue. Throughout her growth dystonia, hiperlaxity of the joints, poor strength, walking disabilities, myopia, absence of verbal skills, and growth retardation were noticed.

### Standard karyotyping

Chromosome analysis was performed on blood lymphocytes, using GTG high resolution banding technique, according to standard procedures [17].

### Array CGH

DNA was extracted from the proband and parent’s peripheral blood, using the QIAmp DNA Mini kit (Qiagen, Valencia, CA, USA) according to manufacturer’s instructions. DNA concentration was determinate with NanoDrop ND1000 spectrophotometer and software (NanoDrop Technologies, Berlin, Germany). Array CGH analysis was performed using the Agilent kit 4×180K (Human Genome CGH Microarray, Agilent Technologies, Santa Clara, CA, USA), with a 17 Kb resolution, according to manufacturer’s protocol [18]. Genomic positions are referred to the Human Genome February 2009 assembly (hg19).

### Multi ligation-dependent probe amplification

Multi ligation-dependent probe amplification was used to confirm oligoarray-CGH results for chromosome 19 in the proband and parents, with commercially available SALSA P347-A1 for microdeletions syndromes (MRC Holland, Amsterdam, Netherlands) and was performed according to manufacturer’s instructions. Amplification products were electrophoresed on an ABI PRISM 3100 Genetic Analyzer and the data obtained analyzed by excel spreadsheet (MRC Holland, Amsterdam, Netherlands) [19].

Written informed consent was obtained for publication and any accompanying images. A copy of the written consent is available for review by the Editor-in-Chief of this journal.

## Abbreviations

CGH: Comparative genomic hybridization
DECIPHER: Database of Chromosome Imbalance and Phenotype in Humans using Ensembl Resources
ISCN: International System for Human Cytogenetic Nomenclature
MLPA: Multiplex Ligation-dependent Probe Amplification
MOR: Minimal overlaping region
OMIM ID: Online Mendelian Inheritance in Man Identification
